# Editorial: Methodological development and applications of nonlinear dynamic analysis for neuroimaging

**DOI:** 10.3389/fneur.2024.1387356

**Published:** 2024-03-14

**Authors:** Donghui Song, Kay Jann, Danny J. J. Wang

**Affiliations:** ^1^The State Key Laboratory of Cognitive Neuroscience and Learning, Beijing Normal University, Beijing, China; ^2^IDG/McGovern Institute for Brain Research, Beijing Normal University, Beijing, China; ^3^Laboratory of Functional MRI Technology (LOFT), Keck School of Medicine, USC Stevens Neuroimaging and Informatics Institute, University of Southern California, Los Angeles, CA, United States

**Keywords:** neuroimaging, nonlinear dynamic analysis, brain entropy, complexity, dynamic causal modeling (DCM), dynamic brain networks, fMRI

The human brain is a complex system with non-linear spatiotemporal dynamics. High-level brain functions emerge from the complex interactions of neurons over a wide range of temporal and spatial scales, and evaluating nonlinear dynamics of the brain's signals offers a new perspective in both normal and diseased conditions. With the ongoing advancements in neuroimaging, there has been an increasing interest and research focus on the application of nonlinear dynamics analysis in recent years. Therefore, this Research Topic, “*Methodological Development and Applications of Nonlinear Dynamic Analysis for Neuroimaging*,” is dedicated to the research of nonlinear dynamic analysis methods and application for neuroimaging. Specifically, the Research Topic delves into brain entropy and complexity, dynamic brain networks, and dynamic causal models (DCM), all within the broader context of nonlinear dynamics analysis.

Complexity metrics such as sample entropy have been extensively applied to the study of various brain functions and diseases, revealing patterns associated with cognitive functions and disorders. Liu et al. applied sample entropy of resting-state fMRI in classical trigeminal neuralgia (CTN) which is a common and severe chronic neuropathic facial pain disorder. They found increased sample entropy in the thalamus and brainstem, and decreased sample entropy in the inferior semilunar lobule in CTN compared to healthy controls (HCs). Moreover, thalamus sample entropy and neuropsychological assessments revealed a significant positive correlation. Liu et al. also used sample entropy as feature to the classification of CTN and HCs using machine learning, and have shown the potential utility of sample entropy alteration as a diagnostic marker for CTN (Liu et al.). Estimation of fMRI complexity can be influenced by many factors, such as signal time scale and sensitivity threshold, as well as the signal variations induced by head movements. Previous studies have made some efforts in understanding and evaluating these potential influences (1–3). In this Research Topic, Roediger et al. proposed an optimized multiscale sample entropy approach using a windowing approach to reduce motion effects and a process for selecting analysis parameters. These findings emphasize the importance of addressing motion for the calculation of multiscale sample entropy (Roediger et al.).

Brain network analysis based on functional connectivity (FC) is widely used in the neuroimaging field and several large-scale brain networks have been consistently and repeatedly identified in the human brain, such as default-mode network (DMN), salience network (SN), and central executive network (CEN). While static FC (sFC) treats the whole timeseries as one state, dynamic FC (dFC) of brain networks provides more detailed information about dynamic state changes. Feng et al. applied sFC and dFC in amnestic mild cognitive impairment (aMCI) and Alzheimer's disease (AD) based on a triple network model, including the above-mentioned networks. Significant differences in sFC and dFC within the triple network were observed, with sFC and dFC capturing distinct characteristics of spontaneous brain activity in HCs, aMCI, and AD (Feng et al.).

Finally, while common fMRI task analysis using general linear models is able to identify brain areas responding to specific task conditions, they are unable to test non-linear interactions and dynamic changes within and between brain areas. DCM, however, facilitate the understanding of effective connections among different brain regions and estimate hidden neuronal states based on measured brain activity. Yang et al. identified the motor cortex, cerebellum, and visual cortex by using a general linear model during the tracking tasks with and without feedback and then employed a DCM and parametric empirical Bayes to quantitatively elucidate the interactions among the left motor cortex (ML), right cerebellum (CBR) and left visual cortex (VL). They found that the tracking task with visual feedback strongly affected the modulation of connection strength in ML → CBR and ML↔VL and the modulation of VL → ML, ML → ML, and ML → CBR by the tracking task with visual feedback could explain individual differences in tracking performance and muscle activity, this study enhances our understanding of the mechanisms underlying human motor control (Yang et al.).

Overall, this Research Topic focuses on the methodological development and applications of nonlinear dynamic analysis for neuroimaging. These studies contribute to expanding the application of nonlinear dynamics analysis in neuroimaging and optimizing existing measurement methods. We anticipate that nonlinear dynamic analysis will serve as a robust tool, accelerating our understanding of complex brain functions and fostering its application in clinical translational research.

## Author contributions

DS: Writing – original draft, Writing – review & editing. KJ: Writing – review & editing. DW: Writing – review & editing.
